# Forty years literature review of primary lung lymphoma

**DOI:** 10.1186/1749-8090-6-23

**Published:** 2011-03-03

**Authors:** Haralabos Parissis

**Affiliations:** 1 Cardiothoracic Dept, Royal Victoria Hospital, Belfast, Northern Ireland

## Abstract

There are several unresolved issues through out the literature regarding the entity of primary lung lymphoma. Extensive literature review of this uncommon pathology is carried out.

By taking into consideration the reported experience, the author discuss the classification, clinical features, histological differential diagnosis, prognostic criteria, therapeutic management and outcome of primary lung parenchyma lymphocytic infiltrates.

## Introduction

Primary Lung Lymphoma (PLL) is a rare entity (0.4% of all lymphomas[1] & 3.6% of non- Hodgkins lymphomas[2]) of heterogenous group of patients with some common characteristics: 1) difficult to be diagnosed due to an indolent course (with a tendency to relapse) with a non specific clinical and radiological presentation 2) low diagnostic yield and 3) sometimes difficult to differentiate from pseudo-lymphomas and 4)overall good outcome especially in disease amenable to surgical resection.

The main diagnostic criterion for PLL is the absence of extra-pulmonary involvement. That means unilateral or bilateral involvement of the lung with or without hilar or mediastinal lumph node involvement and with or without chest wall involvement [3]. Therefore, in patients with biopsy-proven lymphoma of the lung, PLL is diagnosed if extra-pulmonary involvement is ruled out.

In this article we aim to review the literature in order to delineate from the surgeons prospective, the overall experience of the rare entity of PLL and also to bring up to date the variables leading to a favorable outcome following surgery.

## Materials and methods

Pertinent medical literature in the English language was identified through a Medline computerized literature search and a manual search of selected articles using as Key-words: Primary pulmonary Lymphoma, Lung Lymphoma, Pseudolymphoma of the lung, Non-Hodgkin lymphoma of the lung or extranodal lymphoma. The search terms were combined using the Boolean operator term "or" to find all abstracts pertaining to the chosen search terms. These individual terms were then combined using the Boolean operator term "and" to find articles that contained information of all search terms (as per Greenhaligh et al)[4]. The reference lists of articles found through these searches were also reviewed for relevant articles. Links provided on the web sites of published articles were searched for relevant articles. The primary search yielded 81 relevant articles. Of those 14 were excluded as they pertained to pseudolymphoma.

The Ann Arbor pulmonary lymphoma staging system was used for classification

### Stage

IE: Lung only, could be bilateral

II 1E: Lung and hilar lymph nodes

II 2E: Lung and mediastinal lymph nodes

II 2EW: Lung & chest wall or diaphragm

III: lung and lymph nodes below the diaphragm

IV: diffuse

### Characteristics of the various reports

We reviewed the reported literature from 1966 to 2007. We divide the reports into 2 groups. The first group (Table 1[5-62]) contains case reports with fewer patients compare to the second group (Table 2[63-71]).

**Table 1:** Published Studies with small number of patients nHL: Non Hodgkin Lymphoma MALToma: mucosa-associated lymphoid tissue

Author	Year	Journal	Number	nHL	MALT	Comments/Outcome
Gao J[5]	2002	Zhonghua Jie He He Hu Xi Za Zhi	6			Misdiagnosis is common. Persistent cough is the most common symptom

Zhang L[6]	2006	Zhonghua Wai Ke Za Zhi	10	8		3 cases IE, 2 cases II 1E, 2 cases II 2E and 1 case of II 2EW. All patients had Pneumonectomy & ChemoTx. Survival > 17 months. Advanced (stage II 2E) B-cell low grade and Hodgkin disease lead to poor prognosis

Tian XL[7]	2008	Zhonghua Jie He He Hu Xi Za Zhi	18	7	9	CT features: nodules 14/18, Pleural effusion 5/18. Treatment with Surgery & CTx/RTx. Survival > 11 months: 13 pts, with one death and 4 patients lost to follow up

Varona JF[8]	2005	Tumori	6	6		Mono-CTx treatment with alkylating agents. The authors suggest that the outcome is favorable whatever the treatment modalities

Peterson H[9]	1985	Cancer	6	5		Authors suggest that the treatment is surgical resection and that Rtx and CTx are used when residual disease is present after surgery. Median time to death:48,6 months

Muller C[10]	1990	Rev Pneumol Clin	9			Treatment is surgical in localized forms; there is no firmly established treatment in extensive forms

Mu XD[11]	2007	Beijing Da Xue Xue Bao	1		1	MALT with features: consolidation of right middle lobe and left lower lobe, left pleural effusion with monocytes, monoclonal protein in the electrophoresis of serum, CD20 positive

Natali F[12]	1984	Rev Pneumol Clin	2	2		Discussion about PLL related diseases with a varying degree of malignancy: interstitial lymphocytic pneumonitis ILP, pseudolymphoma PL, lymphomatoid granulomatosis LYG

Deng L[13]	2003	Zhonghua Jie He He Hu Xi Za Zhi	3	3		Radiological features of 3 cases and the role of percutaneous biopsy

Nakachi S[14]	2007	Gan To Kagaku Ryoho	2			2 cases of PPHodgkinL

Martinez RC[15]	2004		1	1		PPL presenting as a pulmonary mass with cavitation

Colby TV[16]	1982		20	20?		

Toh HC[17]	1997	Leuk Lymphoma	11	11		Mean age 50. Lower lobe involvement was the commonest. Small lymphocytic lymphoma was the most common. Good symptom control and radiologic response was achieved with chemotherapy

Marchevsky A[18]	1983	Cancer	5			Criteria for pseudolymphoma Vs PLL. 167 Cases in the literature were analyzed

Morisako T[19]	1998	Nihon Kokyuki Gakkai Zasshi	6	6		Southern blot analysis of lung biopsy: rearrangement of a heavy chain gene

Kim JH[20]	2004	Jpn J Clin Oncol	24	9	15	50% of the patients were asymptomatic at presentation. Bronchoscopy: 30% yield, 67% needed surgical procedure for diagnosis. Overall survival at 3 years: 86%

Addis BJ[21]	1988	Histopathology	15			The diagnosis was based in 13 cases: on Light chain restriction

Arinc S[22]	2006	Tuberk Toraks				Review paper on the current approach in PLL

Xu HY[23]	2007	Chin Med J	12		12	Diagnosis and treatment of MALTomas. 2 pts also had gastric MALTS. Operation was performed on 6 patients. 4 pts treated with Chemo alone. Mean survival 71.3 months. One patient experience recurrence 152 months following operation. Several treatment methods can be used to achieve good outcomes

Pagani M[24]	2007	Tumori	1	1		Single case of right hilar LL.

Cao MS[25]	2008	Zhonghua Jie He He Hu Xi Za Zhi	2 cases of NK/T cell L. Also Literature review of 3 cases			Aggressive tumors. Contrary to nHL most patients presents with symptoms. Pleural effusions 4/5. Ebstein-Barr was positive in 3/5. Those tumors are CD56(+), CD3(+) but CD20(-). Most pts died within 6/12.

Baas AA[26]	1986	Eur J Respir Dis	1	1		Single case of a 49 y old man with multiple ill defined densities in both lungs treated successfully with Chemotherapy

Ziade N[27]	2005	J Med Liban	1	1		Single case of PLL in an elderly patient

Habermann TM[28]	1999	Semin Oncol				Review article with an emphasis to observations in the clinical management and treatment of PLL

Uematsu M[29]	1997	Kyobu Geka	1	1		PLL of Rt middle lobe treated with lobectomy

Tillawi IS[30]	2007	Saudi Med J	2			2 cases of P Hodgkin lymphoma in young patients. CD30 and CD15 positive in RS cells were detected.

Chu HQ[31]	2007	Zhonghua Jie He He Hu Xi Za Zhi	13		13	MALT is more common in middle age males. Variable radiographic features; bilateral disease in more than 50% of the cases

Le Tourneau A[32]	1983	Hamatol Oncol	15	15		Reference to Kiel- Lennert histo pathological classification. Association of PLL of B type and dysimmune disease

Loh KC[33]	1994	Ann Acad Med Singapore	3	3		Interestingly, despite nodal involvement all patients had surgical resections and adjuvant ChTx. All 3 alive at 92, 51 and 12 months

Cordier JF[34]	1984	Rev Mal Respir	4			The article raises the possible hypothesis that pseudolymphoma may be the initial step in a large spectrum ranging from benign to malignant primary lymphoproliferative lung disorders

Watanabe J[35]	1987	Jpn J Med	1	1		The diagnostic value of surface marker analysis in primary B cell lung lymphoma is emphasized

Toishi M[36]	2004	Kyobu Geka	2		2	Report of 2 cases of MALToma treated with Surgery and post op RadioTherapy

Jayet A[37]	1980	Helv Chir Acta	10			This report emphasizes the fact that surgical treatment of PLL has to be "economical" due to the fact that frequent recurrences (sometimes bilateral) could be encountered

Kuroishi S[38]	2003	Nihon Kokyuki Gakkai Zasshi	1		1	A case of a lingular lobe PLL that relapsed with diffuse micronodular pattern 7 years following surgical resection

Sakula A[39]	1979	Postgrad Med J	1	1		A single case report

Hashizume T[40]	1997	Nihon Kyobu Shikkan Gakkai Zasshi	1	1		A single case report of PLL presented with bilateral infiltrative shadows

Gouldesbrough DR[41]	1988	Histopathology	1			A single case of PLL diagnosed by bronchial cytology and immunocytochemistry

Bosanko CM[42]	1991	J Comput Assist Tomogr	1	1		A single case report presented as an asymptomatic chronic lobar consolidation

Chee YC[43]	1986	Ann Acad Med Singapore	1			Report of a Pseudolymphoma case with a biclonal gammopathy

Bolton- Maggs PH[44]	1993	Thorax	2		2	Report of 2 cases of MALTomas, giving emphasis on the varied clinical and radiological features

Xu TR[45]	1987	Zhonghua Jie He He Hu Xi Za Zhi	2		2	Report of 2 cases of MALTomas, giving emphasis on the varied clinical and pathological features

Konig G[46]	1986	Prax Klin Pneumol	1			The role of BAL in diagnosis of PLL

Ehrenstein F[47]	1966	J. Thorac Cardiovasc Surg	2		2	2 cases of PLL

Tamura A[48]	1995	Jpn J Clin Oncol	24	24		PLL: relationship between clinical features and pathologic findings Pulmonary LL were divided into 4 groups Bcell tumors composed of small to medium size lymphoid cells have the best prognosis

Sakuraba M[49]	2000	Nihon Kokyuki Gakkai Zasshi	3		2	Report of 3 cases

Abe Y[50]	1998	Nihon Kokyuki Gakkai Zasshi	1		1	One case of MALToma diagnosed with flow cytometer analysis, monoclonal gammopathy and Southern blot analysis of the heavy chain of the immunoglobulin gene

Umino T[51]	1993	Nihon Kokyuki Gakkai Zasshi	1	1		A case of PLL diagnosed with: High serum IgG, BAL showing 45% plasma cells and 18% lympocytes, CD19(+), IgG/albumin ratio 13 times higher and IL-6/albumin ratio29 times higher in BAL than serum. The PCR on the DNA extracted from the surgical specimen showed rearrangement of the immunoglobulin heavy chain gene

Zinzani PL [52]	2003		12			MALTomas

Herbert A[53]	1984	Hum Pathol	9	9		The authors claim that histologic evidence of lymph node involvement is unusual even in the presence of mediastinal or pleural infiltration

Davis WB[54]	1987	Chest	1	1		Report of one case of bilateral interstitial infiltrates with lymhocytic alveolitis on the BAL

Pisani RJ[55]	1990	Mayo Clin Proc	1	1		Report of the first case wherein PLL was diagnosed with immunohistologic (less diagnostic for T cell lymphomas) and molecular biologic studies of BAL.

Sprague RI[56]	1989	Chest	1			A case of an elderly female with multiple densities on CXR. Diagnosis was made with transthoracic fine needle aspiration

Julsrud PR[57]	1978	Radiology				Pseudolymphoma & lymphocytic interstitial pneumonitis have a different radiographic pattern to lymphocytic lymphoma

Lewis ER[58]	1991	AJR Am J Roentgenol	31			CT findings of pulmonary lymphoma: masslike consolidation (68%), multiple nodules (55%). 2/3 of the patients have more than one type of CT finding simultaneously

Bellotti M[59]	1987	Respiration	5			Report a series of 5 PLL out of 9 lymphomas involving the lung

Kilgore TL[60]	1983	Chest	4			4 cases of endobronchial nHL. The authors claim that all the patients had disseminated disease at the time of endobronchial involvement.

Rose RM[61]	1986	Cancer	3			3 cases of endobronchial nHL. The authors have identified 2 patterns of endobronchial involvement: Type 1 characterized by submucosal infiltrates occurring in the presence of disseminated disease and Type 2 whereby the central airway is involved by a solitary mass in the absence of disease elsewhere.

Oka M[62]	1988	Am Rev Respir Dis	1			A case report, whereby the diagnosis of PLL was made 5 years after initial presentation

**Table 2:** Published Studies with large number of patients

Author	Year	Journal	Number of Patients	Characteristics	Appearance	Recurrence	Survival
Koss MN[63]	1983	Hum Pathol	161 14% pseudolymphomas	138 nHL. Elderly, mainly asymptomatic	Most cases: Solitary nodule or infiltrate	Most recurrences occur within 3 years	18 out of 101 patients died from tumor. Pleural effusion was a predictor of mortality

Turner RR[64]	1984	Cancer	47	28 cases of PLL			Good prognosis: 1 patient died in 4 years follow up

L Hoste R[65]	1984	Cancer	36 nHL	Mean age 53 y. More than 80% of patients >40 y	Single lesions 20 cases. Multiple: 16. Unilateral 26, bilateral 10. IE:24, II1E:2, II2E:8, II2EW:2 58% LPI	33% of LPI recur. 50% of non LPI recur. Average time to recurrence:69 months	33% died, most non LPI. No survival difference among cases grouped according to stage(IE Vs II2E). For stage IE LPI group did better. 5 years survival 57%

Kennedy JL[66]	1985	Cancer	64 pts with lymphoid lesions of lung	12 patients with primary lymphoma	Heterogenous group of patients		Median survival of 117 months if PLL. For Disseminated lymphoma median survival 33 months

Li G[67]	1990	Histopathology	62 cases. All B cell but 2 cases of T-cell lymphoma	43 cases of MALT	32 of the MALTS showed solitary or multiple sharply defined nodules	Recurrences in 46% of the MALTs	Constitutional symptoms and T cell lymphoma showed a bad prognosis.

Cordier JF[68]	1993	Chest	70 nHL, no mediastinal adenopathy	Mean age 58.4 y, M:F 1:1, majority non smokers. 87% Low grade. majority MALTs. 13% high grade	Localized opacities 87%. Mass -like appearance 24%, Bilateral disease 21%. Monoclonal gammopathy 30%.	Metastasis 7 pts(stomach, bone marrow, spleen, liver) interval between Dx and mets from 10 months to 7 years	69% underwent surgical resection. Overall survival 93.6% at 5 years for low grade L 26% treated with chemotherapy alone

Ferraro P[69]	2000	Annals of Thorac Surg	48 nHL	MALTs 73%. Mean age 61.8 years. Symptoms 62.5% of patients,	Mass lesion 60%, Bilateral disease 44%. Mediastinal-hilar lymphadenopathy 31%. Pleural effusions 15%. IE: 37 patients, II2E:7, II2EW:3, Stage III:1	Local recurrence 50%	Complete surgical resection 40% overall. Incomplete resection in 29 patients (21 patients with bilateral disease) 73% of MALTs had complete resection. Post op Chemo 54%. Five year survival for MALTs 68% and 10 years 53%..

Graham B[70]	2005	Annals of Thorac Surg	18	MALTs 78%. Mean age 66.4 y M:F 1:2 Symptoms 78% of patients,	Nodules or Mass lesions 72%, Bilateral disease 39%. Mediastinal-hilar lymphadenopathy 39%. Pleural effusions 22%. IIE (39%) pts.	Median time to disease recurrence or death: 6 years	6% died of disease. Five year survival > 80%

Hu YH[71]	2009	Ann Hematol	22	MALTs 54%	Nodules or masses 73%. Mediastinal lymphadenopathy MALTs/non-MALTs: 8/80		Patients who had surgery tended to have better survival. Five year survival MALTs/Non-MALTs 91% over 21%.

## Results

### Group A

58 reports were identified and reviewed. There were 309 cases of PLL. The largest series in this group [58] included 31 patients.

Non Hodgkin Lymphomas (nHL) consist the majority of PLL with mucosa-associated lymphoid tissue (MALTomas) being 70-80%. Hodgkin lymphoma was reported in a small number of cases 1.5-2.4%.

The course of the disease is long and indolent spanning from 1.5 to 108 months [31].

Radiological appearance have shown that non specific patchy opacities or mass-like consolidation was the case in the majority of the patients (up to 68%) and multiple nodules in more than 50% of the cases. [31,57,58].

Nodal involvement (stage II1E, II2E) was reported in 35%-45% of the cases [6,18,58].

Bronchoscopy obtained a diagnostic yield in 30%-40% [20,48] of the cases and invasive surgical procedure revealed the diagnosis in up to 70% of the cases[20].

There was no uniform protocol throughout the literature regarding the indications for surgery. Despite nodal involvement (stage II 1E and 2E) surgery was advocated in few studies [33] and some authors concluded that several treatment methods can be used to achieve good outcomes [23].

### Group B

The second group consisted of 506 reported cases of PLL.

In the majority of the studies the male to female ratio is variable (from 1/1 [68] to 1/2 [70]). Average age of disease presentation is 53±12 years [65,68-70]. 83% of the patients have been reported to be above 40 years of age [65].

Symptoms are present in 2/3 of the patients (62.5% to 78% of patients) [69,70]. The mean duration of symptoms was 5 months [65]. Mild symptoms with no resolving consolidation should be managed with a high index of suspicion.

Again, nHL consist the majority of PLL with MALTomas being 60-78% [69-71].

The radiographic appearance shows nodules or mass lesions in 60-72%,[69,70]. Single lesions are present in 55% of the cases [65]. Multiple nodules are present in 40% of the patients [65,67]. Bilateral disease varies in different reports: 21%. [68], 27%[65], 39% [70], 44% [69].

Nodal involvement (stage II1E, II2E) was reported in 28 to 39% of the cases [65,69] and Pleural effusions 15-22%[69,70].

There was again, no uniform protocol throughout the literature regarding the indications for surgery. Nevertheless, surgery was advocated in 60-70% of the patients. The MALToma patients tend to have complete resections. Broadly speaking, following surgery, patients had more favorable outcome [71].

### Diagnosis

The role of monoclonal protein in the electrophoresis of serum protein: ie. Serum IgG >5000 mgr/dl has been stated in some reports [19,21,51]. Stained for Kappa & Lambda chains, using the immunoperoxidase technique on paraffin sections has been reported. Serum protein electrophoresis abnormalities could be present in up to 33% of the cases [65]. Serum or immunofluorescence monoclonal gammopathy should exclude pseudoL. Furthermore the presence of a serum monoclonal gammopathy is associated with worse prognosis [66].

The significant role of Broncho Alveolar Lavage (BAL) with a cell count of plasma cells of 40% and lymphocytes of 17% with prominence of CD19 positive lymphocytes has been reported by Umino et al [51]. IgG/albumin ratio 13 times higher and IL-6/albumin ratio 29 times higher in lavage fluid than in serum. Furthermore TBLB and immunohistochemical stains mainly CD20 could be helpful. DNA extraction from the surgical specimen and PCR reveals rearrangement band of the genes to the heavy chain immunoglobulin (Fr3a & VLJH primers) [51]. The diagnostic value of Cell surface markers analysis using fresh tissue was also stated in some reports [35,65].

Staging workup should include bone marrow biopsy and CT of the abdomen to exclude extrathoracic disease. Lymphangiograms and bone scans could be part of the preoperative staging [65].

The role of PET scan is equivocal due to the low avidity and the multifocal nature of the disease. The low yield of bronchoscopy and Transcutaneous needle CT guided biopsy has been stated in few reports[13,20]. There is however, a high role for VATS or open surgical lung biopsy with a diagnostic yield more than 90%.

### Histology

PLL arises from centrocyte-like cells normally present in bronchus associated lymphoid tissue. Monomorphic cell population and invasion of bronchial cartilage, pleura or lymph nodes are suggestive of malignancy. Some cases of PLL appeared as complications of a pre existing dysimmune disease (Pigeon breeder disease), Gougerot-Sjogren, Lymphomatoid granulomatosis (LYG) and Liebows lymphomatoid granulomatosis)[12,19,32].

The gross classification of Low Grade malignant Lymphoma higher grade MALTomas (large cell type) and others (ie. Follicular, Diffuse large B-cell, anaplastic large cell)

has been used through out the literature. A detail attempt to estimate the biological potential of lymphomas by their histologic type and correlate this with outcome was attempted with the use of various systems of histologic classification of non Hodgkins lymphomas.

Although from the surgeons prospective the classification seems complicated, one could grossly divide the tumors into small round lymphocytes (50-60%), with varying degrees of plasmacytic change, ("plasmacytoid" well differentiated) and large lymphocytic tumors.

More specifically the Kiel classification [32] essentially differentiates Lymphoplasmacytic (LP) from Centroblastic lymphoma (CB). The LP type is the most common (55-65%) [65]. The tumor is composed of uniform small round lympocytes; they often grow as solid masses; infiltration of bronchial and vascular wall is often however necrosis does not present. The incidence of recurrence is less than 35% through out the literature [65].

Contrary CB type makes up 40% of the cases. It consists of a centrocytic diffuse or follicular population including the rare immunoblastic type. In general those tumors are aggressive, present as pulmonary infiltrates therefore are not amenable to surgical resection and have a higher tendency to recur.

The term MALTomas was described by Bienenstock et al [72]. They are the most common amongst nHL (76% according to Cordier et al). The MALTomas are characterized by: extranodal, small lymphocytic b-cell tumor, cellular heterogeneity, infiltration of the bronchial mucosa by centrocyte cells and presence of reactive lymphoid follicles. The etiology of this mucosal transformation is probably acquired in response to long-term exposure to various antigenic stimuli; Synchronus MALTomaS involving the lung, stomach & ocular adnexa have been reported in the literature [68,70].

Pulmonary lymphomas of nH type could be divided into 4 groups [48]according to the properties and behavior of the tumor: B-cells small or medium size (those tumors are frequently associated with consolidations and air bronchograms), B-cell large lymphoid cell (frequently radiologic presentation is consistent with a mass) and T-cell tumors (bad prognosis). Furthermore the REAL classification sums up the histological variations of all types of lymphomas [73] however, from the surgical prospective it is detailed and probably not widely applicable.

### **Differentiating between Lymphoma (especially Lymphoplasmacytic (LP) type) and pseudolymphoma**

Mixture of mature lymphocytes and plasma cells with reactive follicles are suggestive of pseudolymphoma.

Pulmonary pseudolymphoma is a rare lesion; Up till 1980, only 30 cases were reported in the literature. True lymphoma was developed in four cases [34].

The diagnosis of pseudolymphoma is based on: 1) pulmonary nodules composed of cytologically benign lymphoid cells 2) presence of infiltrates with plasma cells, histiocytes and monocytes 3) presence of germinal centers. In addition, the presence of pleural effusion does not preclude the diagnosis of pseudolymphoma.

Immunological studies aim to define whether the lymphoid proliferation is monoclonal or polyclonal. It is suggested that PPL arises from centrocyte-like cells normally present in bronchus -associated lymphoid tissue. In addition to malignant population reactive follicles and polytypic plasma cells are frequently present so one should be aware that cases previously diagnosed as pseudo-lymphoma or lymphoid interstitial pneumonia need to be reconsidered. Moreover when recurrent tumors are present then pseudo L is rare; however pseudolymphomas do not necessary follow a benign course [18]

### Surgical Treatment strategies

Resection rate varies from series to series [18,37,68,69]. There are no guidelines as to when surgery is indicated. While reviewing the literature one gets the impression that surgery is advocated on an institutional basis.

Surgery for solitary lesions and adjuvant therapies for more extensive disease has been the general consensus. Overall 60-70% of the patients with PLL are surgical candidates [68]; however, incomplete resection is reported to be the case in more than 50% of the cases [69]. Surgical candidate could potentially be any patient with locally resectable tumor up to stage II 2EW. Lymph node involvement does not appear to be a contraindication to surgery. Likewise bilateral disease could also be tackled surgically. However the surgical ablation of such lesions must be economical [37] because of frequent recurrence, sometimes bilateral. Hu et al [71] concluded that patients who had received surgery tended to have a better 5 year overall survival.

The MALTomas are slow growing tumors with an indolent course; tent to be localized and therefore amenable to surgery (73% complete resection was achieved [69]).

Combined modality therapy appears to be superior in patients with bulky disease, residual disease following operation and an unfavorable non-MALT type of histology.

### Recurrence rates

Pulmonary recurrences are either within the ipsilateral lung or in both lungs. Extrapulmonary recurrent disease occurs mainly in lymph nodes, however skin, bone marrow or visceral organs could be affected [63,65].

The overall local recurrence rate is 50% [69]. More specifically the incidence of recurrence for the LP group is 33% with an average time of 69 months versus 50% for the CB group [65].

The median time to disease recurrence or death has been reported to be 6-7 years [70,68]. Late recurrences up to 14 years have also been reported [23].

There are not enough data in the literature regarding Surgery for recurrences; nevertheless the general consensus dictates that recurrences should be treated with aggressive chemotherapy regimes.

### Survival data

This is a heterogenous group of patients. Nevertheless the overall reported median time to death was 7 years or the overall reported mean survival was 71.3 months. More specifically for low grade lymphomas the median survival was 117 months and for disseminated lymphoma 33 months according to Kennedy et al [66].

The overall survival at 3 years was 86% and at 5 years 57% [68,65]. For the MALTomas the five year survival was 68% and the ten year 53% [69].

The prognostic factors influencing survival are: the histologic type, T cell lymphoma [67] the presence of pleural effusion [63] as well as bilateral disease and the need for adjuvant therapy [70]. In contrary according to Ferraro et al [69] complete Vs incomplete resection, the stage of the disease, the presence of mediastinal lumph node involvement or bilateral disease did not significantly influence survival.

## Discussion

Extensive literature review of the medical literature the last 40 years was carried out. We have excluded cases of primary pulmonary AIDS related lymphoma and lymphoma following immuno-suppression and transplantation (200 fold higher than the general population).

The incidence of PLL has two peaks: the first on in the fifth decade of life and the second late six and seventh decade. The diagnostic criteria for PLL include bilateral pulmonary lesions as PPL. The reason for that is the fact that several of those patients when treated never showed evidence of extapulmonary involvement. Also the definition includes absence of extrapulmonary disease for 3 months following the initial diagnosis. This is because invariably extrathoracic and extranodular lymphoma may present (ie. Pulmonary and stomach Lymphoma)

High index of suspicious facilitates the diagnosis of this rare, indolent disease. One third of patients have no symptoms, furthermore out of the symptomatic cohort the duration of symptoms prior to the diagnosis is at least 5 months. Serum protein electrophoresis abnormalities are present in 33% of the patients [65].

The frequency of imagine features of PLL is difficult to assess in the literature because of varying radiological terminology & heterogeneity of several series including all lymphomas of the lung. The radiography is non informative (solitary nodule, multiple ill defined nodules, consolidated mass with air bronchograms, ground glass opacity or reticular lesions in one or both lung fields, pleura effusions, atelectasis and cavities) and non specific. Multiple lesions can be present in up to 25% of the patients [65], air space consolidation with air bronchograms is the most frequent imaging in up to 65%-70% of the cases and pleural effusions in 25% of the cases [31].

The histological classification having kept the principles of low grade (87% of the patients, as per Cordier et al[68]) and high grade disease, has evolved into more complex classifications taking into account the cell morphology and histological characteristics. Although, that does not necessarily correlate with prognosis the gross differentiation into MALTs and non MALTs somehow reflects prognosis[71].

The Staging systems considers bilateral pulmonary lesion as stage I disease and stage II as disease confined to the thoracic cavity;

Only surgical biopsy and resection obtains high yield (64% of the patients as per Cordier et al[68] & 90% of the patients as per Ferraro et al[69].

The prognostic factors affecting survival are not well defined; the stage of the disease, extend of resection (complete Vs incomplete) and the presence of mediastinal lymphadenopathy does is not associated with worse prognosis [69]. In contrary, the report by Hu et al [71] suggested that hilar or mediastinal node involvement negatively influences survival. Higher stage disease was associated with statistically not significantly worse outcome according to Graham et al [70]. The authors reported that bilateral disease was the most significant factor predicting disease recurrence and death.

The evaluations of the role of surgery as well as the indications for surgery are scarce in the literature; positive surgical margins do not alter survival therefore the role of surgery may be applicable in the majority of the cases following by chemotherapy.

The long term outcome of PLL is favorable; 56% of the patients recovered from the disease [70] with an overall 5 year survival across the border of >60% and a recurrence rate of less than 50%.

During the analysis of the presented series we observed a low consistency in publishing specific variables (ie. Incidence of recurrence, etc) and this precluded us from carrying out detailed statistics; therefore our paper carries the biases not only from the studies examined but also from its observational character. Nevertheless, we believe that our report has attempted to give an insight in this rare and not well addressed pathology.

## Competing interests

The authors declare that they have no competing interests.
